# Social Experiences, Discrimination, and Violence among Men Who Have Sex with Men in a Northern Brazilian Capital

**DOI:** 10.3390/healthcare11070964

**Published:** 2023-03-28

**Authors:** Alexandre Mansuê Ferreira Carneiro, Yan Corrêa Rodrigues, Maria Fani Dolabela, Luana Nepomuceno Godim Costa Lima, Ricardo José de Paula Souza Guimarães, Carl Kendall, Ligia Regina Franco Sansigolo Kerr, Karla Valéria Batista Lima

**Affiliations:** 1 Program in Parasitic Biology in the Amazon Region (PPGBPA), State University of Pará (UEPA), Tv. Perebebuí, 2623-Marco, Belém 66087662, Brazil; 2 Bacteriology and Mycology Section, Program in Epidemiology and Health Surveillance (PPGEVS), Evandro Chagas Institute (SABMI/IEC), Ananindeua 67030000, Brazil; 3 Department of Pharmacy, Federal University of Pará, Guamá, Belém 66075110, Brazil; 4 Geoprocessing Laboratory (LABGEO), Evandro Chagas Institute (IEC), Ananindeua 67030000, Brazil; 5 Tulane University School of Public Health and Tropical Medicine, 1440 Canal St, New Orleans, LA 70112, USA; 6 Health Sciences Center, Department of Community Health, Federal University of Ceará, Rua Prof. Costa Mendes, 1608-5th Floor, Rodolfo Teófilo, Fortaleza 60430140, Brazil

**Keywords:** discrimination, MSM, stigma, healthcare

## Abstract

Men who have sex with men who suffer stigmatization and discrimination become more fragile in facing life’s problems, such as the search for treatment in health services. In the present study, the social aspects related to discrimination and violence among men who have sex with men in Belém, Pará, are evaluated. Data were obtained by applying the respondent-driven sampling method to recruit 349 participants aged 18 years or older and who reported having had at least one sexual relationship with a man in the last 12 months. Data were collected from June to December 2016 in a semi-structured interview. Five seeds were initially recruited who applied RDS. The vast majority were between 18 and 35 years old, had completed elementary school but not high school, and were of mixed race. Almost a third lived in peripheral neighborhoods and were employed/self-employed. Additionally, most participants reported having suffered aggression/discrimination, more often in religious contexts, with family or in health services. The findings reported here may contribute to the development of public policies aimed at this population and indicate the need for new strategies to combat sexually transmitted infections, stigma, and discrimination suffered by this population.

## 1. Introduction

Violence in Brazil has been part of nation building, especially the aggression suffered by natives, enslaved black people, and other minority populations with the colonization process contributing to racism and negatively impacting the social ascension of such groups and their descendants [1]. There is still a great socio-economic asymmetry between white and black people in Brazil [2]. Regarding the number of murders among young people, a significant number of victims are black or mixed race and live in the outskirts of large cities and in socio-economic conditions of high vulnerability [3,4]. In this context, it is necessary to emphasize the triple prejudice that people may suffer when they are black/brown, poor, and from the outskirts, which may hinder their access to quality education, better jobs, social ascension, and escape from a discriminatory and violent environment [2,5,6,7].

Another factor that can contribute to discrimination and violence is sexual orientation, especially among men who have sex with men (MSM). This group includes individuals who, regardless of whether they have sex with women, have a hetero social or personal identity, or identify themselves as ‘gay’ or ‘bisexual’, are defined according to their sexual practice and not by gender identification or sexual orientation [8,9,10]. The MSM population and those belonging to the lesbian, gay, bisexual, transgender, and queer (LGBTQIA+) community suffer constant violation of their rights in addition to stigma and discrimination, which can impact their social and professional lives and even their access to health services [11,12,13]. Additionally, HIV serologic status, an infection still detected in higher prevalence in this population, can further aggravate this situation [14].

It is worth mentioning that stigma and discrimination encompass a series of complex factors involving the individual and social spheres. Several studies conducted in a wide range of settings highlight the negative impacts of stigma and discrimination on mental health, education level and school attendance, risky sexual behavior, seeking HIV testing and counseling, and adherence and access to HIV/AIDS treatment [15,16,17,18,19,20,21,22]. 

Surveys among hard-to-reach populations, including MSM, may contribute to understanding and minimizing the negative impact caused by stigma and discrimination. Methods such as respondent-driven sampling (RDS) use a previous selection of seeds representing influential individuals from the MSM environment and various social strata [23]. Here, we describe the application of the RDS methodology to recruit participants for a study evaluating aspects of stigmatization/discrimination and violence faced by MSM in Belém, Pará, as well as the social factors that influence these processes.

## 2. Materials and Methods

### 2.1. Study Design and Logistics

This is a cross-sectional and retrospective study, based on quantitative methods, conducting 350 semi-structured interviews in order to analyze discrimination and violence-related aspects among members of the MSM population in the city of Belém, Pará, Brazil. The study is one of 12 studies conducted as part of a national surveillance study of MSM and HIV in 12 cities in Brazil, also applying previously described methodology [24,25,26,27]. Sample size was determined by the Brazil Ministry of Health. 

After defining the logistics and procedures for carrying out the study, the Marco Health Center School (CSE-Marco) was selected as the site for the implementation of field-related activities, given the familiarity of potential participants with the healthcare unit. The CSE-Marco is a teaching and assistance unit that has basic and specialty care services, with a multi-professional team of physicians, nurses, dentists, psychologists, social workers, nutritionists, occupational therapists, and physical therapists. The CSE-Marco works together with the Center of Biological Health Sciences at the State University of Pará, integrating teaching, research, and community service. The selected healthcare unit was conveniently located for our study population, accessible by public transport, and available from 5 pm to 11 pm, a time period in which most of the participants were available.

### 2.2. Data Collection Strategies and Inclusion Criteria 

Project participants were recruited using respondent-driven sampling (RDS), widely-used for studies with vulnerable and difficult-to-access populations, such as MSM. It is a chain sampling method that begins with the participation of key informants from the population (or seeds) known to the study researchers and the community of interest. Seed inclusion criteria included individuals belonging to non-governmental organizations (NGOs) with active militant behavior in the LGBTQIA+ cause and a large network of contacts who were willing to participate in the study. In this study, three coupons were given to each seed who then started the first wave of their chain by giving their coupons to three new respondents. These new participants also received three coupons, each repeating the previous process until the calculated sample size of 350 MSM participants was reached.

Included in this stage of the study were MSM who were aged 18 years or older; reported having had at least one sexual relationship with a man in the last 12 months; lived, studied, or worked in Belém; and accepted the participation conditions, which included: I. participating in the interview; II. being physically and mentally fit to participate; III. agreeing to invite their peers to participate in the study; and IV. signing the Free and Informed Consent Terms (TCLE). Participants under 18 years of age, those who were under the influence of drugs, including alcohol, at the time of the interview, or who identified themselves as transsexuals or transvestites were excluded from the sample.

Data collection was carried out from June to December 2016 through semi-structured interviews with each participant. In order to reduce potential bias during data collection, audio computer-assisted self-interviewing (ACASI) technology was adopted for the interviews, which were performed using a tablet PC. Prior to interview, the field researchers defined a unique identification number for each participant, preventing identification of individuals and their respective answers. No interview data were accessed by the field researchers. All MSM who met the inclusion criteria were interviewed and answered questions regarding social network, age, education level, ethnicity, marital status, housing situation, religion (type and practice), sexual knowledge and behavior, employment, and family income as well as type, place, and number of times they had suffered aggression/discrimination and violence and reporting the aggression/discrimination suffered.

### 2.3. Data Analysis

Following weighting using RDS-Analyst^®^ data were analyzed using descriptive analysis of the explanatory variables through frequency distribution of the categorical variables and measures of central tendency of the continuous variables. The G-test of independence was applied to verify the association between variables and adjusted residuals were used as a post-hoc test after a statistically significant G-test of independence. Values of *p* ≤ 0.05 were considered statistically significant. All analyses in this study were performed using the statistical software BioEstat^®^ 5.4 [28].

### 2.4. Ethical Considerations

The study was approved by the Research Ethics Committee of the Federal University of Ceará (COMEPE/UFC No 1.024.053) and was carried out in the city of Belém in the state of Pará. Informed consent was obtained from all MSM individuals involved in the study.

## 3. Results

**Figure 1:**
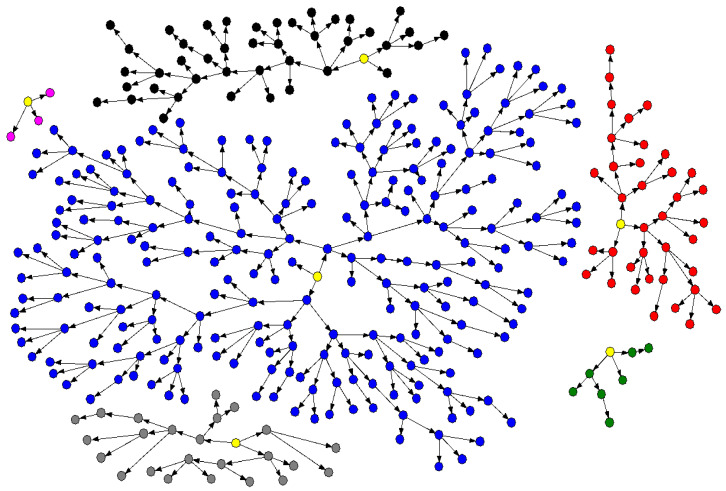
MSM recruitment network in Belém, Pará, Brazil, 2016. Legend: Seeds in yellow originating the networks/clusters represented in black, blue, red, pink, green, and gray.

The initial number of participants was 350, however we had a sample loss of one participant according to the exclusion criteria, and the data analyzed in this study were therefore a total of 349 MSM (Figure 1). Figure 1 shows the recruitment of participants, where the five seeds (represented by the circles in yellow) can be observed as originating different groupings (represented in colors: black, blue, red, pink, green, and gray) totaling the 349 MSM recruited in this study.

All the participants were between 18 and 51 years old, with the majority between 18 and 35 years old (90%) and therefore concentrated in the youngest age group of the population (Table 1). The mean age of the population included in the study was 25.2 years. More than half (71.1%) had completed Elementary II/middle school and a small proportion (9.1%) were in higher education. 

**Figure 2:**
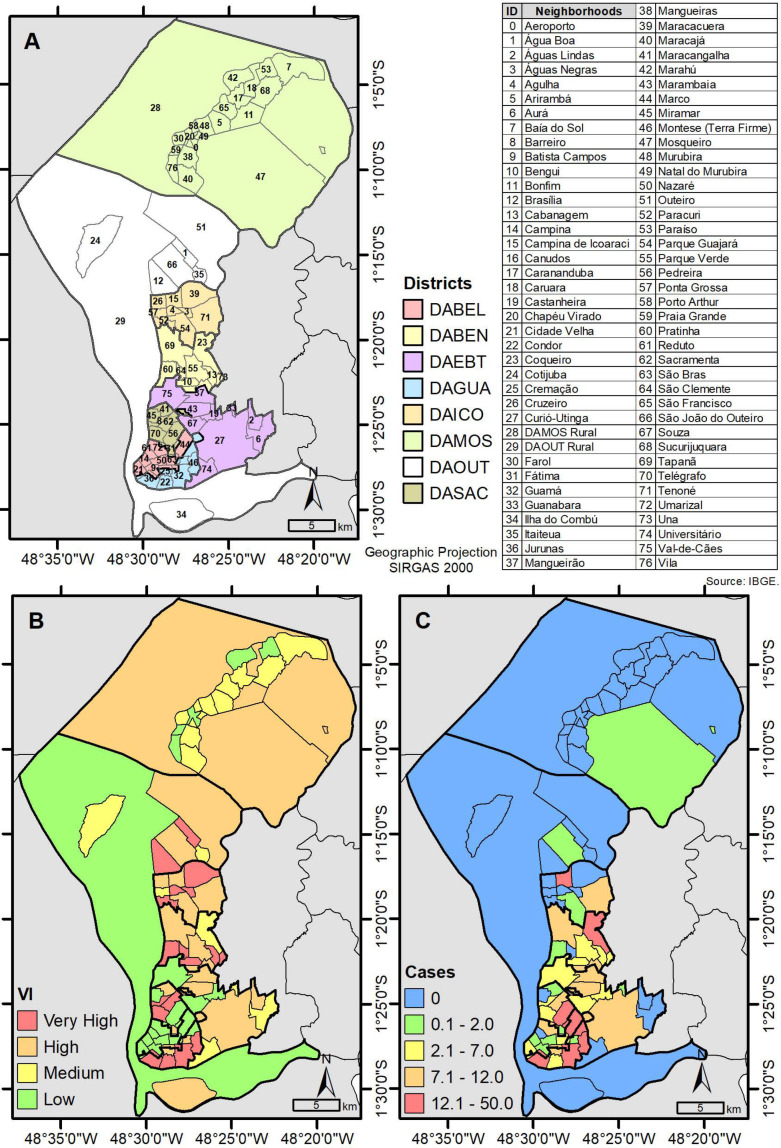
Maps of MSM distribution in neighborhoods by administrative district, Belém, Pará, Brazil. Source: TerraView/IEC/UEPA, 2022. (**A**) location of neighborhoods by administrative district, the (**B**) vulnerability index by neighborhood, and (**C**) the frequency of MSM by administrative region.

In terms of ethnicity, most of the subjects included were brown (61.1%), and most were living in neighborhoods of greater socio-economic vulnerability in the city of Belém (Figure 2). In that same figure, we can also observe the location of neighborhoods by administrative district (A), the vulnerability index by neighborhood (B), and the frequency of MSM by administrative region (C).

Regarding the marital status of the MSM in this study, there was a predominance of single subjects (79.4%). A large portion (97.6%) stated that they lived with family members in their own house (42.8%), and more than 80% of the interviewees were not head of the household (Table 1). The majority declared they identified with a religion (79%) with about half practicing it (51%). The majority were Catholic (44.4%), followed by Evangelical/Protestant (Table 1). 

This research shows that 90.8% of the participants had an income of up to two times the minimum wage. When evaluating family income, 76.3% had incomes of up to BRL 2000.00 in the year 2016 (Table 1). Regarding work, a relatively low percentage (28.9%) had a steady job, and participants also reported temporary work (8.3%), self-employment (29.5%), and unemployment (23.4%). Among the unemployed, 87.8% reported not being able to obtain a job (Table 1).

Most of the MSM participants in this study had experienced some type of aggression/discrimination (79%) at least once (8.8%) or up to eight times (6.3%) (Table 2). The participants had been discriminated against at work, with some (14.9%) not being selected for a job or being fired. Others reported being poorly served or prevented from entering businesses/leisure places (21.9%) as well as in health services or by health professionals (14.9%) (Table 2).

In educational environments, the MSM in this study reported that they had been mistreated or marginalized by teachers at school/college (23.6%) and also by friends (33.4%), neighbors (36.3%), and family (38.7%), in religious environments (30.8%), and at blood donation clinics (23.6%). The police station was also an environment of discrimination (21.9%) as well as participants being badly attended to or badly treated in public services, such as at hostels, subprefectures, transportation, or public bathrooms (16.7%) (Table 2). In addition, some (14.4%) reported having been blackmailed or suffered money extortion. At some point, many participants had felt afraid to walk in public spaces (43%) (Table 2). The majority (54.1%) had communicated their discrimination at least once, often to family members (18%) but also to other people, such as the police, lawyers, social networks, relatives’ acquaintances, and justices (Table 3).

In relation to the ethnicity, education, and family income of those who had reported aggression/discrimination, we can observe that more than half declared themselves to be brown (61.1%), followed by white (16.8%) and black (16.5%), that most had completed Elementary II but not middle school, and that most had a family income of between BRL 100.00 and 1000.00. There was also a higher frequency of people with an income of up to one minimum wage who had suffered aggression (Table 4).

## 4. Discussion

MSM who suffer stigmatization are perceived to become more fragile in facing everyday problems, such as the search for treatment in health services. In this context, the Ministry of Health, taking into consideration the social and structural determinants of disease created the National Policy for Comprehensive Health for Lesbians, Gays, Bisexuals, Transvestites, and Transsexuals to seek better equity in the provision of services for these groups [29,30]. Among the MSM population, there is still low demand for adequate information, health services, and prevention methods on the part of those living with HIV/AIDS, mainly due to the fear of being stigmatized and/or discriminated against. The fear of raising suspicions about their serological status means this group tends not to adopt behaviors that may contribute to reducing the risk of infection by STIs [14].

In the present study, predominantly younger people (18–35 years old) were included. It is known that in Brazil, on average, sexual life begins between 13 and 17 years of age, thus, precociously [31]. Besides the physiological issues, it is necessary to evaluate if young people are prepared to deal with the discriminatory and stigmatizing process that they may suffer. At this stage, individuals dealing with prejudice and discrimination may become more vulnerable to mental disorders [32]. The first sexual intercourse often occurs during adolescence, a time when the individual does not yet have a set of conditions (emotional and/or knowledge) to allow him to handle situations that may bring consequences to his health or lacks the maturity to adopt good practices of prevention of STIs and AIDS [33,34].

In this study, a very worrisome situation is related to the participants’ education, which was predominantly equal to or less than high school (Table 1). Low education can also be related to low-income conditions, causing a negative impact on access to basic services, especially health services [35]. Thus, evidence has indicated that advancing in schooling increases the likelihood of accessing better jobs, increasing income, and improving socio-economic conditions [36]. However, even today, members of the LGBTQIA+ community are victims of prejudice in the school environment, which can lead them to drop out of school [37]. National survey data from the U.S. highlight several deficiencies in the educational context, which remains biased, sexist, and unsafe for LGBTQIA+, and mainly for those of color [17]. Similarly, as observed in Brazil, 1,016 elementary school students over the age of 13 anonymously answered a questionnaire revealing that 73% had suffered homophobic bullying; 60% felt unsafe at school; and 37% had already suffered physical violence. In the seven countries studied (Brazil, Argentina, Chile, Uruguay, Peru, Colombia, and Mexico), the data were very similar, with the exception of Uruguay where all rates are below 50% [38,39,40]. Therefore, making schools more inclusive through educational campaigns aimed at clarifying sexuality and gender-related issues can contribute to improving this scenario [40,41,42].

Regarding the civil union of MSM, most of the interviewees reported being single, a fact that may be related to later marriage, as found in other studies [30]. Many times, young people opt for liquid relationships, where there is no commitment and the person does not matter; only pleasure is sought at any cost and sex is only an instrument of pleasure [43,44,45,46]. In a society of liquid relationships, subjects are increasingly anxious, sad, and emotionally overloaded.

Another important issue observed in the present study was that most respondents reported residing with relatives. The incorporation of changes in moral tolerance by society has enabled families to allow their children’s “stayers” to visit and even share the same room, leading children to extend their stay in their parents’ home [47]. 

In terms of ethnicity, most participants self-declared as brown or mixed race. According to IBGE’s National Continuous Household Sample Survey, 95.9 million Brazilians identify as brown, representing 46.7% of the total Brazilian population. In the North Region, where the municipality of Belém is located, more than half of the population (72.3%) declared themselves brown, 19.5% white, and 7% black, explaining the presence of a higher percentage of this group in this study. At this point, it becomes important to discuss the double prejudice that these people can experience, being both MSM and brown or black [48]. Thus, we can say that these are stigmas that intersect and interact because as well as suffering from the stigma of a non-normative sexuality, these individuals suffer because of their race/ethnicity. Furthermore, when these stigmas are associated with a serological condition, in the case of HIV, they act synergistically, making them even more difficult to face and also causing impacts on public health [49]. 

The participants included in this research lived in neighborhoods of greater socio-economic vulnerability in the city of Belém. According to health professionals and managers, in addition to prejudice, living in the periphery is also a barrier faced by MSM to access HIV testing and treatment, for example [9]. In the realm of economic and social determinants, these are decisive factors in influencing the health conditions of individuals and populations [50]. 

The association of these stigmatizing factors can hinder access to jobs, and may also be one of the reasons related to the difficulties of obtaining a new job by respondents. A study conducted in 2016 showed that the unemployment rate in the general Brazilian population was 9.6%, that is, lower than the unemployment rate observed in the population of this study. A cause that can partially explain this high unemployment rate is the low education of the subjects included in the research as well as their ethnicity, residing in the periphery, and also sexuality issues [5].

In cases of racism, when reported to the police, the aggressor can be arrested, and this should also occur in cases of discrimination against someone because of their sexual identity or orientation. Although there are already laws dealing with this subject, a lack of attention is still observable when prejudice is suffered by people from the LGBTQIA+ community [51]. 

There are several types of violence suffered by MSM, and the consequences for the victim’s health can be varied. People who are victims of discrimination may be eight times more likely to commit suicide, six times more likely to report depression, three times more likely to use licit and illicit drugs, and three times more likely to engage in unprotected sex [52].

The majority of this study’s participants declared having a religion. Since the colonization of Brazil, homosexuality has been related to sin, and this fact is related to the Christian religion. Prejudice in relation to sexual identity may be related to the remoteness of people in the sacred spaces of each religion, and this may be one of the reasons for not practicing religion [53]. 

According to international human rights law, the stance of neutrality regarding the social, economic, political, and cultural conditions of human beings was abandoned. In this regulatory framework, it has been admitted that certain individuals and groups are in a situation of vulnerability and inequality and require differentiated treatment, including in legal terms [54]. In this context, it is important to evaluate the places where discriminatory processes may be occurring in this study. There is a need to understand the types of discrimination that a person may be subjected to, including direct (intentional) and indirect (unintentional) discrimination. Among the manifestations of this discrimination, three stand out: institutional, invisibility of privilege, and neglect. In the field of this work and in other scenarios, perhaps indirect discrimination occurs more often, and it may not be perceived by the victim. However, it is very important to discuss the consequences of this type of discrimination for the life of MSM. This fact may contribute to the rate of 19.9% of MSM who responded that they had not experienced discrimination [55].

Other studies have observed that the stigma and discrimination that individuals in this population suffer increase in countries of medium and low income as compared to those with high income [16,17,18,19,56,57,58,59,60]. In this same discussion, we can consider the hypothesis that individuals who belong to the MSM population end up being socially marginalized in multiple social and cultural contexts [52]. The scenario observed in Belém, Pará, is permeated by an association of factors that may predispose MSM to stigma and various types of violence since most individuals fit a socio-epidemiological profile of vulnerability, including being of brown/black ethnicity, having low education, and living in conditions of high socio-economic vulnerability, that contributes to stigma and prejudice [2,52].

Despite the methodology used in this study (RDS sampling) allowing the collection of a huge amount of information in a short time period, there are limitations in this technique, such as potential selection biases and other assumptions associated with RDS. Individuals were recruited through social networks and the period/time in which individuals were subjected to discrimination. As in all survey research, there can be a lack of motivation for subjects to properly answer the interview as well as to provide false answers to sensitive topics. 

## 5. Conclusions

Through this local analysis, the present study findings call for future research and programs to confront violence, stigma, and discrimination against the MSM population, in addition to contributing to the formulation of health, preventive, and intervention policies to promote the reduction of any type of vulnerability of this population in Brazil.

Because the onset of sexual discovery is an important predictor of future care, as well as schooling, we identify the need for early sexual and gender education in schools, especially for young MSM who have not yet had their first sexual experience. However, in the national context, sexual and gender education still suffers attacks from conservative groups. This finding highlights that knowledge about sexual health and gender is necessary to reduce the incidence of STIs and the stigma and discrimination that this population may suffer.

## Figures and Tables

**Table 1:** Sociodemographic characteristics of men who have sex with men in Belém, Pará, Brazil, 2016.

Variables	Nº (%)
**AGE GROUP (years)**	
18–20	102 (29.1)
21–25	136 (38.8)
26–35	80 (22.8)
36–50	28 (8.0)
≥ 51	3 (0.85)
**EDUCATION/SCHOOLING**	
Illiterate/Elementary I incomplete	12 (3.4)
Elementary I complete/Elementary II incomplete	56 (16)
Elementary II complete/middle school incomplete	249 (71.1)
High school complete/higher education incomplete	32 (9.1)
**ETHNICITY**	
White	59 (16.8)
Yellow	9 (2.57)
Brown (Pardo)	214 (61.1
Indigenous	6 (1.7)
Black	58 (16.5)
**MARITAL (CIVIL) STATUS**	
Married	10 (2.8)
Single	278 (79.4)
Separated/divorced	4 (1.14)
Homosexual stable union	53 (15.1)
**HOUSING**	
Rented	49 (14)
With friends	3 (0.85)
With friends/others	20 (5.7)
With parents	117 (33.4)
Own place	150 (42.8)
Rented room	9 (2.5)
No fixed address	1 (0.28)
**HAVE A RELIGION**	
Yes	276 (79.0)
No	65 (18.6)
**PRACTICE YOUR RELIGION**	
Yes	178 (51.0)
No	90 (25.7)
**RELIGION**	
Catholic	155 (44.4)
Spiritist/Kardecist	8 (2.2)
Evangelical/Protestant	81 (23.2)
Adventist	1 (0.28)
Buddhist	1 (0.28)
Agnostic	2 (0.57)
Christian	1 (0.28)
Jehovah’s Witness	1 (0.28)
Wicca	2 (0.57)
African Matrix Religion	23 (6.5)
**PARTICIPANT’S INCOME**	
<1 minimum wage (BRL 880.00)	177 (50.7)
1–2 minimum wage	140 (40.1)
2–3 minimum wage	11 (3.1)
3–4 minimum wage	11 (3.1)
>4 minimum wage	10 (2.8)
**FAMILY INCOME**	
BRL 100.00–BRL 1000.00	203 (58)
BRL 100.30–BRL 2000.00	64 (18.3)
BRL 2096.00–BRL 5000.00	62 (17.7)
BRL 5300.00–BRL 10,000.00	13 (3.7)
BRL 10,520.00–BRL 22,000.00	7 (1.98)
**SOURCE OF INCOME**	
Employee with monthly salary	101 (28.9)
Retired/age/length of service	2 (0.57)
Temporary work with salary	29 (8.3)
Autonomous	103 (29.5)
Benefit/sick leave	1 (0.28)
Unemployed	82 (23.4)
University/student scholarship	5 (2.85)
Intern	3 (0.85)
Support from the family	4 (1.4)
No source of income/student	2 (0.57)
**HOUSEHOLDER**	
Yes	38 (10.8)
No	287 (82.2)
**WHO DO YOU LIVE WITH**	
Male partner	30 (10.8)
Female partner	2 (0.57)
Friends	21 (6)
Father-mother	215 (61)
Relatives	100 (28.6)
Male partner/friends	3 (0.85)
Male partner/father-mother partner	6 (1.7)
Relatives/father-mother	28 (8)

**Table 2:** Characteristics related to aggression/discrimination suffered by men who have sex with men in Belém, Pará, Brazil, 2016.

Variables	Nº (%)
**SUFFERED AGGRESSION/DISCRIMINATION**	
Yes	277 (79)
No	67 (19.9)
Did not answer	5 (1.4)
**TYPE OF AGGRESSION/DISCRIMINATION SUFFERED**	
Physical and sexual assault	5 (1.4)
Physical aggression	4 (1.1)
Sexual assault	15 (5)
Discrimination and aggression	40 (14.4)
Discrimination	129 (46.5)
Discrimination and sexual assault	43 (15.5)
Discrimination, physical and sexual assault	45 (16.2)
**NUMBER OF TIMES DISCRIMINATED AGAINST**	
Once	31 (8.8)
Twice	25 (7.16)
Three times	23 (6.5)
Four times	22 (6.3)
Five times	21 (6)
Six times	23 (6.5)
Seven times	18 (5.1)
Eight times	22 (6.3)
**WHERE YOU SUFFERED DISCRIMINATION**	
Not selected or dismissed from a job	53 (14.9)
Poorly served or prevented from entering commerce/leisure places	77 (21.9)
Poorly served in health services or by health professionals	53 (14.9)
Mistreated/marginalized by teachers at school/college	83 (23.6)
Excluded or marginalized from groups of friends	118 (33.4)
Excluded or marginalized from neighborhood groups	127 (36.3)
Excluded or marginalized in a family environment	136 (38.7)
Excluded or marginalized in a religious environment	108 (30.8)
Prevented from donating blood	83 (23.6)
Mistreated by police or poorly treated in police stations	77 (21.9)
Blackmailed or money extorted	51 (14.4)
Poorly attended to or treated poorly in public services	59 (16.7)
**NUMBER OF AGGRESSION REPORTS**	
Not reported	9 (2.5)
Once	189 (54.1)
Twice	39 (11)
Three times	17 (4.8)
Four times	2 (0.57)
Six times	1 (0.28)
Did not answer	92 (26.3)

**Table 3:** Reporting the discrimination experienced among men who have sex with men in Belém, Pará, Brazil, 2016.

Reporting/Communication the Aggression Suffered to Someone		
**Variables**	**YES**	**NO**
Relatives	63 (18%)	194 (55.5%)
Spouse/partner	19 (5.4%)	238 (69.1%)
Friend	133 (38.1%)	124 (35.5%)
Healthcare professional	3 (0.8%)	254 (72.7%)
Precinct	10 (2.8%)	254 (72.7%)
Educational institution professional	14 (45%)	243 (69.6%)
Others *	6 (1.7%)	251 (71.9%)

* Others: Police; lawyer; social networks; acquaintances of relatives; justices.

**Table 4:** Relationship of aggression/discrimination with ethnicity, education, and family income of men who have sex with men in Belém, Pará, Brazil, 2016.

Variables			
**EDUCATION/SCHOOLING**	**SUFFERED AGGRESSION/** **DISCRIMINATION**	**NOT SUFFERED AGGRESSION/** **DISCRIMINATION**	**P**
Illiterate/Elementary I incomplete	8	3	0.4442
Elementary I complete/Elementary II incomplete	39	14	
Elementary II complete/middle school incomplete	204	42	
High school complete/higher education incomplete	26	6	
**PARTICIPANT’S INCOME**			
<1 minimum wage (BRL 880.00)	152	23	0.0065
1–2 minimum wage	100	36	
2–3 minimum wage	8	5	
3–4 minimum wage	9	0	
>4 minimum wage	8	2	
**ETHNICITY**			
White	49	8	0.5614
Yellow	8	1	
Brown (Pardo)	168	42	
Indigenous	3	2	
Black	46	8	

## Data Availability

All relevant data are presented within the manuscript.
